# Association caryotype 47XYY et déficit en 5 alpha réductase révélée par un micropénis: à propos d’un cas et revue de la littérature

**DOI:** 10.11604/pamj.2020.36.48.8209

**Published:** 2020-06-01

**Authors:** Nestor Ghislain Andzouana Mbamognoua, Fatima Aziouaz, Suzanne Matali, Hanane El Ouahabi, Farida Ajdi

**Affiliations:** 1 Service d’Endocrinologie, Diabétologie et Maladies métaboliques, CHU Hassan II de Fès, Fès, Maroc; 2 Équipe Sciences des Médicaments-Centre Médical de Recherche Biomédicale et Translationnelle, Faculté de Médecine et de Pharmacie de Fès, Fès, Maroc

**Keywords:** Caryotype 47XYY, déficit en 5 alpha réductase, micropénis, dihydrotestostérone (DHT), Karyotype 47XYY, 5-alpha reductase deficiency, micropenis, dihydrotestosterone (DHT)

## Abstract

Les sujets 47XYY ont souvent un fonctionnement gonadotrope normal, l’association à un déficit en 5alpha réductase chez ces sujets est rare ; la présentation clinique classique des déficits en 5 alpha réductase est un pseudohermaphrodisme masculin, rarement un micropénis comme mode de révélation. Le traitement par énanthate de testostérone du micropénis ne donne pas de bons résultats dans les déficits en 5alpha réductase, la dihydrotestostérone(DHT) à une efficacité prouvée dans ce cas. Nous rapportons l’observation d’un patient de 17 ans, référé dans notre formation pour la prise en charge d’un micropénis ne répondant pas aux 2 cures à base d’énanthate de testostérone. Le bilan notait une testostérone normale, des gonadotrophines à la limite supérieure de la normale, une DHT basse, avec augmentation du rapport testostérone/DHT > 20.caryotype 47XYY. Le déficit en 5 alpha réductase chez ces sujets pose la problématique d’une simple coïncidence, ou d’un lien effectif.

## Abstract (en)

Subjects with 47XYY often have normal amounts of gonadotropin-releasing hormone. In these subjects the association between 47XYY and 5-alpha reductase deficiency is rare. The common clinical manifestation of 5-alpha reductase deficiency is male pseudohermaphrodism, rarely it has been revealed by micropenis. Testosterone enanthate does not give good results in patients with 5-alpha reductase deficiency; dihydrotestosterone (DHT) has proven effectiveness in these cases. We report the case of a 17-year old patient, referred to our Hospital with micropenis. The patient didn’t respond to two enanthate testosterone therapies. Assessment showed normal testosterone levels, amounts of gonadotropin-releasing hormone at the upper limit of normal, low DHT, elevated testosterone/DHT ratio>20, karyotype 47 XYY. This study highlights that 5-alpha reductase deficiency in these subjects raises the issue of simple coincidence or effective link.

## Introduction

La formule chromosomique 47XYY est une anomalie rare, représentant 1/1000 naissances masculines [1, 2]. Elle se définit par la présence d’un chromosome Y supplémentaire au sein d’un caryotype masculin. Des cas d’épilepsie, de troubles de comportement et d’apprentissage ont été rapportés chez les sujets 47XYY [3], d’autres encore n’ont aucune anomalie décelée rendant le diagnostic difficile. La présence de ce chromosome Y supplémentaire est due à une défaillance de la division cellulaire de la mitose postzygotique dans le développement embryonnaire précoce ou à une non-disjonction paternelle à la méiose II [4,5]. Le micropénis défini par une verge de taille réduite, plus petit que 2.5 déviation standard (DS) en dessous de la moyenne, avec une incidence de 1,5 sur 10 000 garçons nés entre 1997 et 2000 aux USA [5, 6], constitue une circonstance de découverte fréquente des hypogonadismes centraux et périphériques en particulier des anomalies chromosomiques telles que le syndrome de Klinefelter (47XXY), d’un défaut d’action de la testostérone entre autre par déficit en 5 alpha réductase. Le tableau typique du déficit en 5 alpha réductase est un pseudohermaphrodisme masculin (DSD XY), avec un phénotype féminin, rarement un micropénis isolé. L’association d’un caryotype 47XYY et d’un micropénis a été décrite par quelques auteurs [2], la singularité de cette association repose sur le fait que sujets porteurs de caryotype 47XYY ont souvent un fonctionnement gonadotrope normal. L’association à un déficit en 5 alpha réductase est peu décrite. Nous rapportons à travers notre patient, la particularité de la présentation clinique de ce déficit en 5 alpha réductase, et l’association peu fréquente à une anomalie chromosomique 47XYY.

## Patient et observation

**Figure 1:**
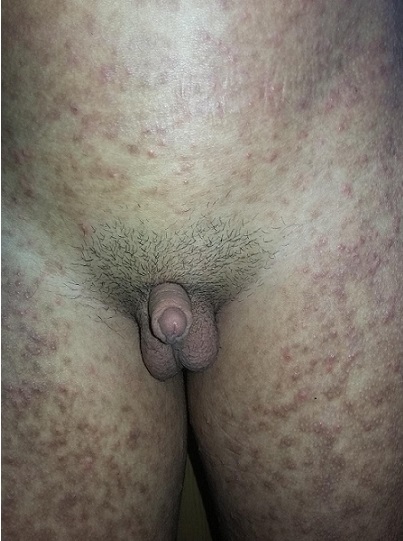
Aspect du micropénis à 3.5cm < -2.5DS, chez le patient 47XYY

**Figure 2:**
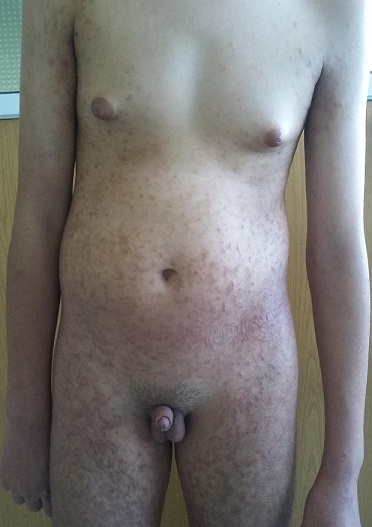
Aspect de la gynécomastie bilatérale stade 2, chez le patient 47XYY

Il s’agit d’un patient de 17 ans, de milieu social défavorisé, aux antécédents de retard des acquisitions motrices dans son enfance, suivi en dermatologie pour une insensibilité à la douleur, des ulcérations secondaires aux automutilations et un pemphigus vulgaire, puis par un endocrinologue privé à l’âge de 14 ans pour verge de petite taille ayant bénéficié de 2 cures de micropénis à base d’énanthate de testostérone à dose correcte par voie intra musculaire sans amélioration significative de la taille de la verge (gain d’1cm), ni d’érection; le patient a été adressé dans notre formation à l’âge de 17 ans. A l’examen clinique, on notait une absence de retard staturo-pondéral, un micropénis à 3.5cm < -2.5DS (Figure 1), une gynécomastie bilatérale stade 2 (Figure 2), sans écoulement mamelonnaire, testicules à 3.5cm (stade 3 de Tanner), pilosité pubienne stade 2 de Tanner. L’exploration des fonctions hépatique et rénale était normale. Le bilan hormonal notait un taux de testostérone totale normal à 5.80ng/ml, les gonadotrophines étaient à la limite supérieure de la normale: FSH = 6.22mUI/ml (0.5-5.3),LH = 5.86mUI/ml (2.6 - 6.2), un taux de prolactine = 13.54ng/ml, l’œstradiol < 10pg/ml, les autres axes endocriniens hypophysaires étaient normaux. Le caryotype est 47XYY. Le dosage de la dihydrotestostérone plasmatique(DHT) concomitamment à celui de la testostérone totale, note une valeur basse de la DHT=0.20ng/ml (0.33-1.20) avec un rapport testostérone/DHT = 29 (> 20) permettant de poser le diagnostic de déficit en 5 alpha réductase. Les échographies testiculaire et abdominale étaient normales. L’étude génétique a été réalisée. Un traitement à base de dihydrotestostérone à application locale a été proposé pour le micropénis et pour la gynécomastie, la condition sociale n’a pas permis au patient de s’en procurer.

## Discussion

Le déficit en 5-alpha réductase est une pathologie rare entraînant chez les patients un pseudo-hermaphrodisme masculin, défini par une différentiation incomplète des organes génitaux chez un patient de caryotype XY [7]. Cette enzyme catalyse la conversion de testostérone (T) en dihydro-testostérone (DHT), hormone indispensable à la différenciation masculine des organes génitaux externes et au développement du tractus uro-génital masculin [8]. La 5-alpha-réductase est codée par le gène SRD5A2. Plusieurs mutations du gène SRD5A2 ont été rapportées responsables du déficit en 5 alpha réductase, situées dans l'ensemble des 5 exons de ce gène localisé en 2p23. Il s'agit en majorité de substitutions d'acides aminés, mais également de délétions, de mutations non-sens [7,9,10]. Sa présentation clinique la plus classique est un pseudohermaphrodisme masculin avec un phénotype féminin à la naissance et une virilisation en période pubertaire [10,11]; notre patient présente un phénotype masculin avec un micropénis et une gynécomastie, qui sont des manifestations, certes décrites du déficit en 5 alpha réductase [12], mais peu fréquentes. Le diagnostic de déficit en 5 alpha réductase est posé devant l’élévation du rapport testostérone/DHT (>20) comme rapporté par de nombreux auteurs [7-12], ce qui est le cas chez notre patient qui avait un rapport testostérone/DHT = 23; cependant le phénotype masculin pourrait s’expliquer par un déficit probablement partiel en 5 alpha réductase. L’étude génétique à la recherche d’une mutation du gène SRD5A2 a été réalisée permettant de confirmer le diagnostic de déficit en 5 alpha réductase chez notre patient. Le caryotype réalisé devant un micropénis associé à une gynécomastie, à la recherche d’une dysgénésie gonadique en particulier le syndrome de klinefelter (47XXY), a révélé chez notre patient un caryotype 47XYY, dans lequel le fonctionnement gonadotrope est le plus souvent normal.la problématique d’une relation de causalité entre le caryotype 47XYY et le déficit en 5 alpha réductase, ou d’une simple coïncidence se posait chez notre patient. De nombreux troubles ont été décrits chez les sujets 47XYY, notamment des troubles moteurs et du comportement retrouvés chez notre patient ont été rapportés dans la littérature [2]. L'étude de la variation neuroanatomique dans 47XYY a montré une augmentation de volume de matière cérébrale, responsable de la fréquence accrue de l'autisme, des troubles du langage avec comme substratum anatomique la réduction de la substance blanche dans le 47XYY [13, 14]. Notre patient ne présentait pas ces anomalies. Des troubles du développement sexuel à type d’hypospadias, de micropénis, d’ectopies testiculaires, associés à un hypertélorisme ont été décrits dans de rares cas.

Le mécanisme, non communément admis, serait l'excès de gènes en raison du supplément de chromosome Y qui pourrait affecter le développement sexuel [15], parce qu’il existe contrairement des sujets 47XYY qui ont un fonctionnement gonadotrope normal, par conséquent une puberté normale. Cet excès de copies de gènes dans le bras long du chromosome Y, des microdélétions Yq sont incriminés dans la survenue de l’infertilité chez les sujets 47XYY, par oligospermie avec des testicules atrophiques [1, 15, 16]. Le profil biologique souvent retrouvé est une baisse de l’inhibine B avec un taux de FSH élevé [15]. Chez notre patient l’absence de réponse à la testostérone exogène, notamment l’absence d’érection spontanée ou après traitement, n’a pas permis la réalisation d’un spermogramme. Le dosage d’inhibine B n’a pu être réalisé faute de moyens financiers. Cependant notre patient avait un taux de FSH élevé laissant supposer une atteinte simultanée de la fonction exocrine du testicule, qui pourrait engager ainsi le pronostic de la fertilité chez notre patient. L’administration de la testostérone sous forme d’énanthate de testostérone en intra musculaire(IM), ou percutanée pendant l’enfance et l’adolescence constitue un traitement efficace du micropénis. Plusieurs protocoles ont été proposés [17, 18] avec des disparités sur les doses, la voie d’administration ou la durée du traitement; Guthrie *et al.* [17] ont proposé une dose de 25 mg d’énanthate de testostérone en IM administrée toutes les trois semaines pendant trois mois., cependant ces études rapportent toutes, une augmentation de la taille de verge sous testostérone exogène. La réponse au traitement par testostérone est considérée comme favorable par certains auteurs, s’il y a une augmentation de 100% de la taille de la verge [19] ou un gain de 3.5cm par d’autres auteurs [18]. Notre patient ne répondait pas au traitement par énanthate de testostérone, correspondant au profil des patients ayant un déficit en 5 alpha réductase. Pour ce type de patient l’application de topique 5-a dihydrotestostérone (DHT) Gel au niveau de la région péri scrotale, trois fois par jour pendant 5 semaines s’accompagne d’une augmentation de la taille de la verge par amélioration des taux sériques de DHT. Chez notre patient, l’incertitude sur l’efficacité du traitement demeurait du fait du diagnostic tardif du déficit en 5 alpha réductase, à l’orée de période d’âge adulte où l’efficacité du traitement médical demeure incertaine du fait de la diminution physiologique des récepteurs aux androgènes (testostérone et DHT), la chirurgie de reconstruction pénienne pourrait s’avérer utile comme autre recours thérapeutique [20]. L’administration de la DHT chez notre patient a permis une augmentation de la taille de la verge à 6cm au bout d’un mois de traitement par DHT.

## Conclusion

Le micropénis est source d’un retentissement psychologique important; il peut être dû à un déficit en 5 alpha réductase, dont le traitement repose sur la DHT. Les anomalies génitales retrouvées chez les patients 47XYY ouvrent la porte d’une recherche permettant une explication précise, tant l’hypothèse de la multiplicité des gènes ne se vérifie pas chez ces patients 47XYY ayant un fonctionnement gonadique normal.

## Conflits d’intérêts

Les auteurs ne déclarent aucun conflit d'intérêts.
